# Both high intratumoral microvessel density determined using CD105 antibody and elevated plasma levels of CD105 in colorectal cancer patients correlate with poor prognosis

**DOI:** 10.1038/sj.bjc.6600874

**Published:** 2003-04-29

**Authors:** C Li, R Gardy, B K Seon, S E Duff, S Abdalla, A Renehan, S T O'Dwyer, N Haboubi, S Kumar

**Affiliations:** 1 Department of Pathology, The University of Manchester, Manchester, UK; 2 Department of Molecular Immunology, Rosewell Park Cancer Institute, Elm & Carlton Streets, Buffalo, NY 14263, USA; 3 Department of Surgery, Christie Hospital, Manchester, UK; 4 Department of Immunology, The Hospital for Sick Children, University of Toronto, Toronto M5G 1X8, Canada; 5 Department of Pathology, Trafford General Hospital, Manchester, UK

**Keywords:** angiogenesis, soluble CD105, microvessel density (MVD), TGF*β*

## Abstract

CD105 and its ligand transforming growth factor *β* (TGF*β*) are modulators of angiogenesis, which drives tumour growth and metastasis. Tumour microvessel density (MVD) has proven to be an important determinant of prognosis. In this study, we have examined the prognostic value of MVD identified using Mabs to the pan-endothelial marker CD34 and to CD105 in 111 patients with colorectal cancer. The Mab to CD105 preferentially reacts with angiogenic endothelial cells. Of the 111 patients studied, 38 were alive and 73 had died of the disease. The median MVD values counted using anti-CD34 and anti-CD105 were 5 (range 1.40–9.00) and 3.10 (range 0.90–8.00), respectively. Kaplan–Meier survival analysis revealed that only MVD values obtained using CD105 Mab correlated with survival. Patients with a high MVD, above the median (3.10), showed the worst prognosis. A similar outcome was observed when MVD was divided into quartiles. In order to ascertain if this strong expression of CD105 in the tumour vasculature is reflected in patients' plasma, circulating levels of CD105, TGF*β*1 and TGF*β*3 together with the receptor–ligand complexes were quantified in patients with colorectal carcinoma and normal controls. Results showed that except for TGF*β*1, the levels of all other molecules were significantly elevated compared with controls. The levels of CD105 were positively correlated with Dukes' stages. A lower TGF*β*1 level was noted in patients with carcinoma over the controls. Furthermore, TGF*β*3 and CD105/TGF*β*3 complexes were markedly lowered in postoperative compared with preoperative plasma samples. Immunostaining revealed that TGF*β*1 was expressed in cancer cells but TGF*β*3 in the stromal cells, whereas CD105 was exclusively expressed in vascular endothelial cells of tumour blood vessels. In conclusion, this study demonstrates that MVD quantified using a Mab to CD105 is an independent prognostic parameter for survival of patients with colorectal cancer, and that plasma levels of CD105, TGF*β*1, TGF*β*3 and CD105/TGF*β* complexes may be useful markers for assessing disease progression. These data have led us to propose that quantification of these determinants may prove useful to monitor therapeutic efficacy in patients with colorectal cancer, especially those who are being treated with antiangiogenic therapies.

Tumour growth and spread are absolutely dependent on their ability to induce angiogenesis (Folkman, 1996). Following the initial publication by Srivastava *et al* (1988) on human melanomas, several subsequent studies have shown an inverse correlation between high tumour microvessel density (MVD) and prognosis in patients with many different types of cancer (de Jong *et al*, 2000; Weidner, 1999, Weidner, 2000). However, some authors have failed to find such a correlation or paradoxically, particularly in colorectal cancers, have observed that high MVD is associated with good prognosis (Chung *et al*, 1996; Abdalla *et al*, 1999). We have proposed that the reasons for these contradictory results may be the use of different antibodies to endothelial cells (ECs), the counting methods and the staining procedures including antigen retrieval techniques (Wang *et al*, 1994). To obtain MVD data, invariably, previous studies have used pan-endothelial antibodies, for example, against von Willebrand factor, CD31 or CD34, which although ideal for staining normal blood vessels, in our experience are inefficient in recognising angiogenic EC. In contrast, CD105 (endoglin) is abundantly expressed in angiogenic EC, and antibodies to it preferentially bind to EC of angiogenic tissues (Krupinski *et al*, 1994; Wang *et al*, 1994; Burrows *et al*, 1995; Bodey *et al*, 1998; Kumar *et al*, 1999; Brewer *et al*, 2000; Fonsatti *et al*, 2001; Akagi *et al*, 2002). Our hypothesis is that this selective immunostaining by an antibody to CD105, that is, its ability to distinguish tumour-associated EC and pre-existing vessels, will reduce the incidence of false-positive staining of normal entrapped vasculature in a cancerous mass (Thompson *et al*, 1987). In two previous studies, we have reported the prognostic significance of MVD identified by Mab to CD105 and of soluble CD105 in the circulation of patients with breast cancer (Kumar *et al*, 1999; Li *et al*, 2000b). Whether this is true for patients with colorectal cancer had not been verified. Therefore, in the present study, we evaluated the prognostic significance of MVD in tumour tissues and soluble CD105 in the plasma collected from patients with colorectal cancer. The MVD was determined using an Mab to CD105, which is highly reactive with angiogenic blood vessels, and a pan-EC antibody, CD34. Both TGF*β*1 and TGF*β*3 bind to CD105 and their functions are regulated by CD105 (Fonsatti *et al*, 2001). TGF*β*1 and TGF*β*3 are also critical growth factors in tumorigenesis. Here, we have determined MVD using a pan-endothelial marker and CD105 in colorectal cancers and have quantified plasma levels of TGF*β*s (viz., 1 and 3) and their receptor, CD105 and receptor–ligand complexes. These data were correlated with other clinical parameters. The results indicate that these molecules may be of potential value as markers of angiogenesis in colorectal cancer patients, who are being treated by antiangiogenic therapies.

## MATERIALS AND METHODS

### Patients

The source of paraffin-embedded sections from 111 patients, with primary colorectal tumours with a minimal follow-up time of 5 years, was the same as previously reported (Table 1

**Table 1:** Some clinical data and their prognostic significance in 111 colorectal cancer patients

**Parameter**	**No. of cases**	**Prognostic significance**
Median age	69.5 years (range 32–95)	NA
		
*Sex*		
Male	65	NS
Female	46	(*P*=0.5140)
		
*Site of tumour*		
Colon	83	NS
Rectum	28	(*P*=0.5537)

*Tumour stage*		
Dukes' A	8	*P*=0.0002
Dukes' B	58	
Dukes' C&D	45	

*Tumour grade*		
Well differentiated	13	NS
Moderately differentiated	61	(*P*=0.4450)
Poorly differentiated	9	
Mucinous	24	
Unknown	4	

*Lymph node*		
Involved	44	
Not involved	67	*P*=0.0004

*Current status*		
Dead	73	NA
Alive	38	

**Figure 1:**
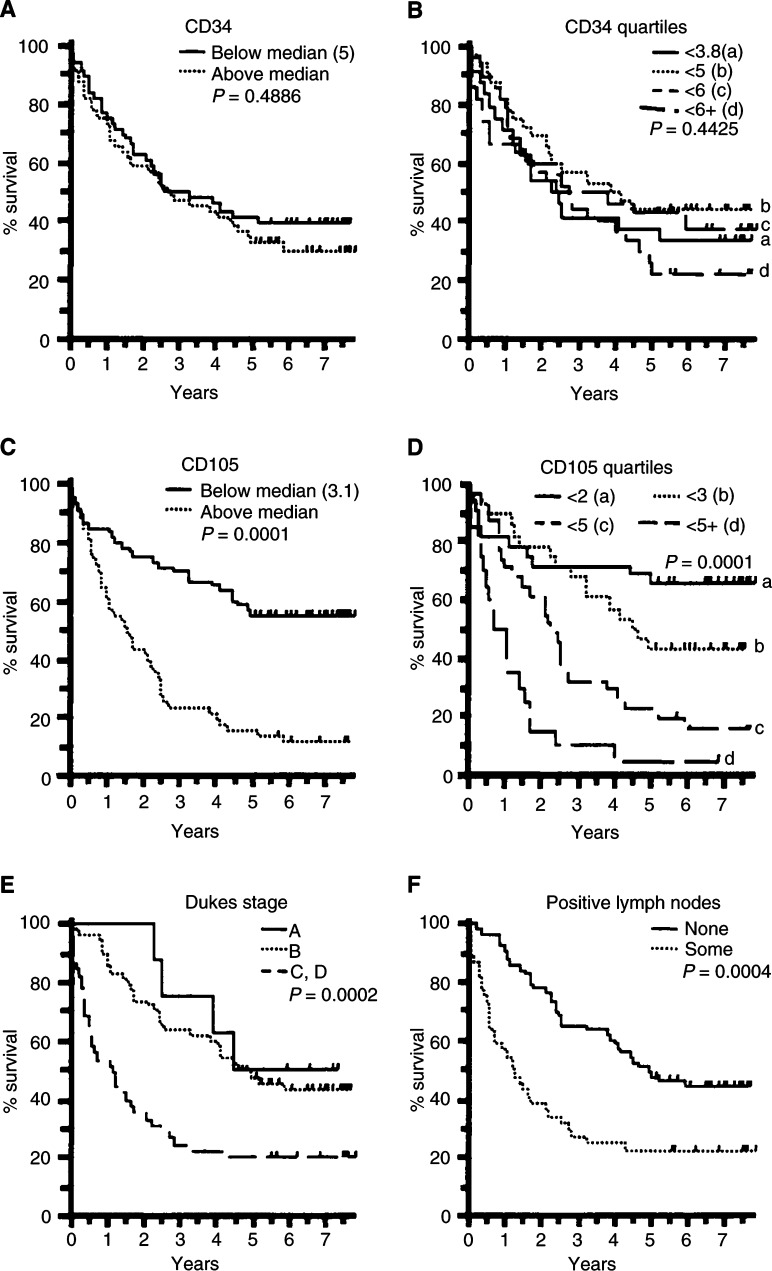
Kaplan–Meier survival graphs showing percent survival of patients with colorectal cancer. Microvascular density data obtained using Mabs to CD34 and CD105 were divided into above and below median (**A**, **C**) and into quartiles (**B**, **D**). Microvessel density values obtained using CD105 showed that high microvessel counts indicated poor prognosis, but there was no significant correlation with MVD values given by CD34. In addition, patients with Dukes' stage C and D (combined) (**E**) or positive lymph nodes (**F**) survived the shortest time. The *P*-values were obtained by log-rank tests.

NS=not significant; NA=not applicable; the *P*-values represent the prognostic significance referred to the survival data in Figure 1 (panel E, F).

) (Abdalla *et al*, 1999). The histopathological parameters of each tumour were ascertained by an experienced pathologist (NH) and assigned as per Dukes' staging system. The overall survival time was 5.60 years (range 0.30–8.00), while the median survival time for those still alive was 6.70 years (range 5.10–8.00 years). Since no plasma samples were available from these patients, a new cohort comprising 76 patients with colorectal cancer and 40 normal controls were recruited. Clinical details of the colorectal cancer patients are given in Table 2

**Table 2:** Clinical data of the 76 colorectal carcinoma patients

*Median age*	67.4 year (range 31–93)
	
*Sex*	
Male	39
Female	37
	
*Site of tumour*	
Colon	38
Rectum	38
	
*Dukes' stage*	
A	8
B	22
C	11
D	35

. Blood samples from patients with colorectal cancer were obtained prior to any treatment. In addition, blood samples were collected from 14 colorectal cancer patients 2 weeks after surgery. Control samples from 40 subjects were collected from healthy hospital staff or from individuals hospitalised for colonoscopy. Exclusion criteria for all control subjects were malignancy, acute or chronic liver and kidney diseases, connective tissue disease, psoriasis, scleroderma, diabetes and symptomatic vascular diseases. Plasma was harvested following centrifugation at 2000 **g** for 10 min at 4°C, aliquoted and stored at −70°C for analysis. Ethics Committee approval and informed consent from all patients were obtained.

### Immunochemical staining of CD105 and CD34

Procedures for the immunostaining of CD34 and CD105 were the same as those published previously (Seon *et al*, 1997; Abdalla *et al*, 1999). Tissue sections were deparaffinised and treated with 3% hydrogen peroxide to quench endogenous peroxidase activity, microwave-treated in citrate buffer and then stained with antibody to CD34 (1 : 100; QBEND-10; Serotec Ltd, Oxford, UK). Incubation with biotinylated secondary antibody was followed with peroxidase-conjugated streptavidin. The colour was developed with diaminobenzidine tetrachloride, and finally sections were counterstained with Mayer's haematoxylin. Negative controls consisted of those sections where primary antibody was omitted. CD105 was stained using mab, SN6 h (Seon *et al*, 1997; Kumar *et al*, 1999). All the slides were scanned to determine the most vascularised areas, that is, ‘hot spots’. Usually the most vascularised areas were located at the tumour periphery. The MVD was determined using a Chalkley eyepiece graticule (× 100 magnification). The mean of four Chalkley counts for each tumour was calculated and used in the statistical analysis.

### Immunohistochemical staining of TGF*β*1 and TGF*β*3

Staining for TGF*β*1 and TGF*β*3 has been described previously (Ghellal *et al*, 2000). Briefly, 5 *μ*m thick cryostat sections were air dried, fixed in cold acetone and then incubated with chicken anti-TGF*β*1 (1 : 400 in PBS; R&D systems) or goat anti-TGF*β*3 (1 : 250 in PBS; R&D systems) overnight at 4^o^C. The sections were washed three times with PBS and incubated with biotinylated anti-chicken (1 : 250 in PBS) or anti-goat (1 : 125 in PBS) antibody in 1% goat serum for 30 min. The horseradish peroxidase (HRP)-conjugated streptavidin (1 : 100 in PBS; Vector) was applied for 30 min and staining was revealed by 0.02% diaminobenzidine (DAB, Sigma) and 0.3% hydrogen peroxidase in distilled water. The sections were counterstained with Mayer's haematoxylin.

The levels of TGF*β*1 and TGF*β*3 expression were semiquantified by light microscopy without knowledge of the patients' details. The staining intensity was scored as negative (−), moderately positive (+) or strongly positive (++).

### Quantification of CD105, TGF*β*1, TGF*β*3 and the receptor–ligand complexes

The immunoassays for CD105, TGF*β*1, TGF*β*3 and the receptor–ligand complexes have been described elsewhere (Li *et al*, 2000a). Briefly, for the detection of TGF*β*1, 96-well white plates (Dynatech Microfluor, VA, USA) were coated at 4°C overnight with 100 *μ*l well^−1^ mouse Mab against TGF*β*1, TGF*β*2 and TGF*β*3 (Genzyme, MA, USA) at 1 *μ*g ml^−1^ in PBS. After blocking with 1% BSA in 0.1 M PBS and 0.1% Tween 20 (PBS–Tween) for 2 h at room temperature, plates were washed three times with PBS–Tween. To release the mature TGF*β*1, plasma was treated using pH 2.0 buffer as described by Grainger *et al* (1995). The acid-activated samples were then transferred to the coated plate. A standard curve was generated using purified recombinant human TGF*β*1 (R&D Systems, Abingdon, UK). The plate was left at 4^o^C overnight in a humidified chamber. Subsequently, the wells were incubated with 100 *μ*l polyclonal chicken anti-TGF*β*1 antibody (R&D Systems, Abingdon, UK), diluted 1 : 1000 (1 *μ*g ml^−1^) in PBS–Tween, for 3 h at 4°C. Three washes with PBS–Tween were given between each procedure. After washing, the plates were incubated on a shaker with 100 *μ* l well^−1^ of rabbit anti-chicken IgG conjugated to HRP (Jackson ImmunoResearch Laboratories Inc. PA, USA), at 1 : 2000 dilution (0.2 *μ*g ml^−1^) in 1% BSA and PBS–Tween, for 30 min at room temperature. Finally, the plates were rinsed three times, 100 *μ*l Amerlite signal reagent (Amersham, UK) was added to each well, and the plate was read immediately in an Amerlite plate reader (Kodak Clinical Diagnostics, Aylesburg, UK). All the samples were run in duplicate and the measured values of light emission at 420 nm were converted into absolute TGF*β*1 concentrations by reference to the standard curve.

TGF*β*3 was quantified using the same procedure as for TGF*β*1, with the exception that the chicken anti-TGF*β*1 was substituted with goat anti-TGF*β*3 (R & D systems, Abingdon, UK) and the untreated plasma was used for TGF*β*3 quantification.

Soluble CD105 was measured using an indirect sandwich ELISA (Li *et al*, 2000b), wherein purified Mab E9, which specifically reacts with CD105, and biotinylated Mab E9, in conjunction with streptavidin peroxidase, were used as capture and detection reagent, respectively. White microtitre plates were coated with Mab E9 (100 *μ*l well^−1^) diluted 1 : 1000 (1 *μ*g ml^−1^) in 0.1 M PBS and incubated overnight at 4^o^C. The coated plates were blocked using 1% BSA and PBS–Tween for 2 h at room temperature. Test samples diluted 1 : 2 in PBS–Tween were added to the plates in duplicate. One plasma sample with a high level of CD105 (approximately 100 ng ml^−1^) was titrated to make a standard curve in each plate. After incubation overnight at 4^o^C, 100 *μ*l well^−1^, biotinylated Mab E9 (1 : 2000 dilution; 0.2 *μ*g ml^−1^) was added to the plates and incubated at 4^o^C in a humidified box for 3 h, followed by addition of 100 *μ*l well^−1^ of streptavidin conjugated to HRP at 1 : 2000 dilution in PBS–Tween and 1% BSA, which was incubated, with shaking, for 30 min at room temperature. Three washes with PBS–Tween were carried out between each of the procedures. Finally, 100 *μ*l well^−1^, Amerlite signal reagent (Amersham) was applied to each well and the light emission at 420 nm was immediately measured in an Amerlite plate reader.

The procedure for quantifying soluble CD105/TGF*β*1 complex was the same as described in the assay for TGF*β*1, with the exception that the coating anti-TGF*β* antibody was substituted by Mab E9 at 1 *μ*g ml^−1^ in order to capture the complex from the serum. A serum sample with a high level of CD105/TGF*β*1 complex (100 arbitrary units ml^−1^) was serially diluted from 1 : 2 to 1 : 512 to generate a standard curve on each plate. The assay showed no crossreaction with CD105/TGF*β*3 complex and exhibited a wide range of detection from 0.05 to 100 Uml^−1^. In the assay for CD105/TGF*β*3 complex, Mab E9 was used as the coating antibody to capture the complex from the plasma. The other procedures were the same as described in the assay for TGF*β*3. The standard curve was generated using a plasma sample containing 50 U ml^−1^ of the complex on each plate.

The sensitivity of the assays for CD105, TGF*β*1, TGF*β*3, CD105/TGF*β*1 and CD105/TGF*β*3 complexes was 0.10 ng ml^−1^, 10 pg ml^−1^, 30 pg ml^−1^, 0.05 U ml^−1^ and 0.03 U ml^−1^, respectively.

### Statistical analysis

Statistical analysis was performed using Mann–Whitney and Kruskal–Wallis tests of central location, Spearman's correlation and survival analysis using log regression and Kaplan–Meier graphs with log-rank tests. CD34 and CD105 were treated as continuous ratio variables, although they were divided into quartiles for the purpose of survival analysis with Kaplan–Meier graphs. Their association with other parameters such as age or tumour size was measured with Spearman's correlation coefficient. Medians of CD34 and CD105 were tabulated for groups of each categorical variable, such as stage. Differences between the medians were tested using the Mann–Whitney or Kruskal–Wallis tests. Kaplan–Meier graphs and log-rank tests were used to determine the prognostic value of the MVD data for overall survival. Multivariate models (Cox proportional hazards) were applied to determine their prognostic value relative to standard factors such as stage. Greenwood's confidence intervals identified how survival differed between quartiles.

For plasma levels of CD105, TGF*β*1, TGF*β*3 and the complexes, results were expressed as mean±s.e.m. and median (range). Differences between groups (viz, controls *vs* carcinoma; preoperative *vs* postoperative patients) were determined using nonparametric Mann–Whitney U-test. Correlation analysis was performed using Kendall's tau_b test. A *P*-value of ⩽0.05 was considered significant.

## RESULTS

### Microvessel density

**Figure 2:**
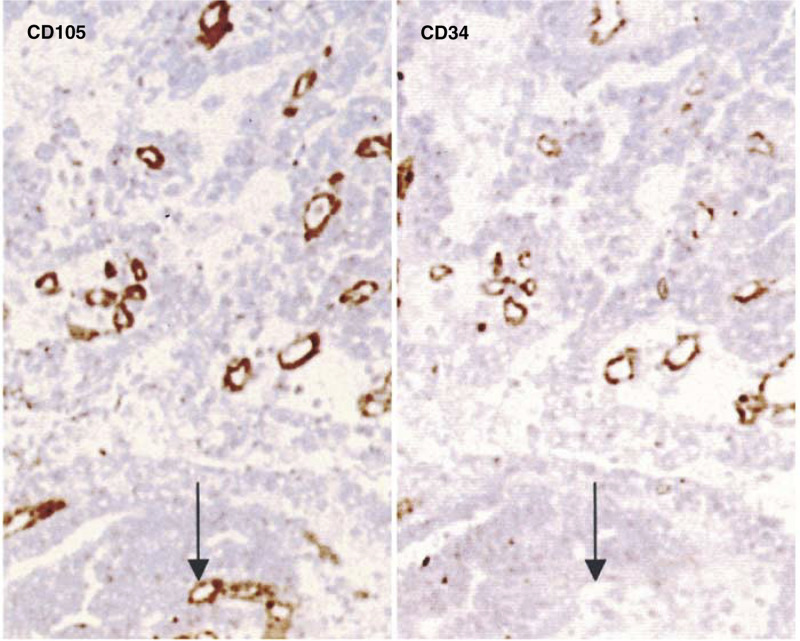
Expression of CD105 and CD34 in tumour vasculature. Serial sections of colon carcinoma were stained with Mabs to CD105 or CD34. Although the majority of blood vessels were stained by both Mabs, a proportion of the vessels reacted only with CD105 but not with CD34 (
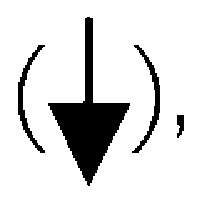
), demonstrating the distinctive expression of CD105 and CD34 in tumour vasculature (× 250).

Of the 111 patients, 73 died – Dukes' stage C and D combined patients had the shortest survival time and positive lymph nodes indicated poor survival (Figure 1 E, F

and Table 1). Survival was not significantly affected by grade, site, patient's age or gender. As shown in Figure 2

, staining for both CD34 and CD105 was heterogeneous. The most highly vascularised areas were invariably observed at the tumour periphery and in the immediate vicinity of normal tissues. The median MVD values for CD34 and CD105 were 5 (range 1.40–9.00) and 3.10 (range 0.90–8.00), respectively. Of the 111 tumours stained with CD34, 56 had microvessel counts below the median, and 55 above the median. The corresponding numbers for CD105 above and below the median were 60 and 51, respectively. The MVD values were also divided into quartiles (CD34: <3.80, <5.00, <6.00 and 6.00+, and CD105: <2.00, <3.00, <5.00 and 5.00+). The survival for patients based on these data is shown in Figure 1 A–D.

Kaplan–Meier survival analysis indicated that MVD obtained using CD34 showed no significant correlation with survival. The same result was noted when MVD was divided by either median value (Figure 1A) or by quartiles (Figure 1B), demonstrating that microvessel counts using CD34 were not correlated with prognosis. In sharp contrast, high MVD counts determined using CD105 were strongly associated with a poor prognosis (Figure 1 C, D). The 5-year survival rate of patients with MVD greater than the median (3.10) was less than 20%, while it was 60% in those with MVD below 3.1 (*P*=0.0001). An inverse correlation between MVD and survival time was also observed when MVD counts were divided into quartiles. Approximately 65% of the patients survived more than 5 years in the group with MVD less than 2. A high MVD recognised by CD105 indicated poor prognosis: patients with MVD greater than 5 lived for the shortest time (Figure 1D). MVD values obtained using CD105 showed no statistically significant correlation with values obtained with CD34. There was no significant correlation between Dukes' stage or lymph node involvement with MVD values given by either CD34 or CD105 (data not shown). CD105 and a panel of other prognostic values were tested for survival using Cox proportional hazard models. Briefly, it emerged that only CD105, lymph node involvement and Dukes' stage were significant as independent prognostic variables. Combined statistics (multivariate analysis) showed that CD105 was an independent prognostic factor for survival. In multivariate models, the prognostic significance of a variable depends on the prognostic significance of MVD values using Mab to CD105 and *vice versa*. Thus, CD105 remained significant whatever variable is added to the model.

### Expression of TGF*β*1 and TGF*β*3 in tumour tissues

**Figure 3:**
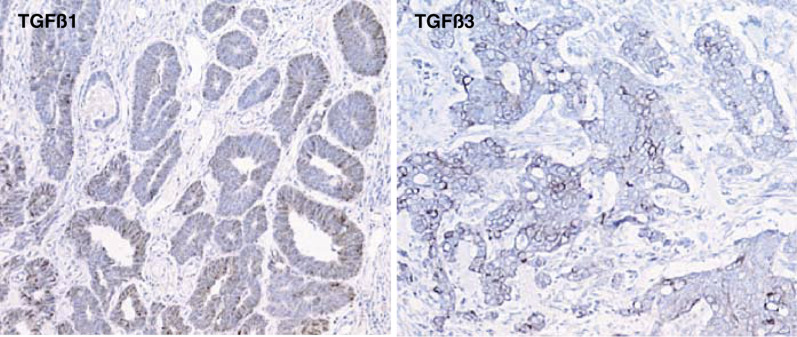
Expression of TGF*β*1 and TGF*β*3 in tumour tissues. Colon carcinoma tissue sections were stained with antibody to TGF*β*1 or to TGF*β*3. TGF*β*1 was localised in the cytoplasm of the tumour cells. In contrast, the expression of TGF*β*3 was mainly in the stromal cells (× 250).

Immunohistochemical staining of TGF*β*1 and TGF*β*3 in tumour tissues revealed their distinctive expression pattern. TGF*β*1 was expressed exclusively in tumour cells, while TGF*β*3 was expressed mainly in tumour stromal tissues (Figure 3

). The expression intensity of TGF*β*1 and TGF*β*3 was analysed for patients' survival using Cox's proportional hazard model. Neither TGF*β*1 nor TGF*β*3 was significantly correlated with overall survival (*P*=0.2740 and *P*=0.6990, respectively). Further analysis revealed no significant correlation with other clinical parameters.

### Plasma levels of CD105, TGF*β*1, TGF*β*3 and the receptor–ligand complexes

The results for the molecules quantified in the plasma are summarised in Table 3

**Table 3:** Plasma levels of CD105, TGF*β*1, TGF*β*3, and CD105/TGF*β* complexes for patients with colorectal carcinoma and controls

**Groups**	**CD105 (ng ml^−1^)**	**TGFβ1 (pg ml^−1^)**	**TGFβ3 (ng ml^−1^)**	**CD105/TGFβ1 (U ml^−1^)**	**CD105/TGFβ3 (U ml^−1^)**
Controls	0.92±0.17	181.80±25.05	0.34±0.06	0.60±0.16	0.04±0.02
*N*=40	0.70	118.60	0.23	0.00	0.00
	(0–3.45)	(67.80–278.00)	(0.05–1.52)	(0–2.70)	(0–0.36)


Carcinoma	3.46±1.30	34.44±4.69	1.54±0.44	1.79±0.24	1.30±0.33
*N*=76	1.89	30.00	0.400	1.200	0.41
	(0–100.00)	(0–207.00)	(0–21.80)	(0–12.50)	(0–16.45)
	(*P*=0.0001) ^*^	(*P*=0.0001) ^*^	(*P*=0.0460) ^*^	(*P*=0.0010) ^*^	(*P*=0.0001) ^*^

Preoperative	2.07±0.28	43.07±14.56	0.59±0.08	1.35±0.28	0.48±0.08
*N*=14	2.01	29.00	0.44	1.15	0.46
	(0.59–4.55)	(0–205.00)	(0.19–1.10)	(0–4.50)	(0.08–1.16)

Postoperative	2.97±0.39	27.87±7.90	1.20±0.72	3.76±1.51	3.40±3.02
*N*=14	3.00	23.13	0	0.80	0
	(0.70–5.30)	(0–114.00)	(0–8.23)	(0–16.90)	(0–42.54)
	(*P*=0.1130) ^**^	(*P*=0.5300) ^**^	(*P*=0.0080) ^**^	(*P*=0.8890) ^**^	(*P*=0.0080) ^**^

Data are expressed as mean±s.e.m. and median (range). The *P*-values obtained by nonparametric Mann–Whitney *U*-test for controls *vs* carcinoma and preoperative *vs* postoperative groups are indicated by ^*^ and ^**^, respectively.

. CD105 was detected in 95% (72 out of 76) of the colorectal cancer patients in contrast to 80% (32 out of 40) in the normal controls. Significantly elevated CD105 levels were seen in cancer patients compared with controls (*P*=0.0001). Furthermore, a positive correlation was observed between CD105 levels and the Dukes' stages, for example, patients with advanced cancer possessed higher CD105 levels than those with early-stage disease (*r*=0.20, *P*=0.0470). The lack of a significant difference between pre- and postoperative samples may be indicative of postoperative angiogenesis during the process of wound healing, which is likely to overshadow a lowered CD105 level following removal of the tumour.

With regard to TGF*β*1, lower levels were seen in patients with cancer compared with controls. It neither differed in pre- and postoperative samples nor was correlated with a particular Dukes' stage. Correlation analysis revealed an inverse correlation between TGF*β*1 levels and the number of positive lymph nodes (*r*=−0.48, *P*=0.0110), that is, those patients with elevated TGF*β*1 levels possess fewer positive lymph nodes than those with lower TGF*β*1 levels. The levels of CD105/TGF*β*1 complexes were significantly increased in patients with cancer, and were inversely correlated with node involvement (*r*=−0.26, *P*=0.0330).

TGF*β*3 was detected in 79% (60 out of 76) of patients with colorectal cancer. The levels of TGF*β*3 in both groups of patients were significantly elevated compared with controls. Comparison of TGF*β*3 levels in pre- and postoperative samples showed a significantly lower level in the latter (*P*=0.0080). However, TGF*β*3 levels were increased in the two postoperative patients, an indication of possible relapse or tumour metastasis (Li *et al*, 1998). The same trend was noticed with CD105/TGF*β*3, that is, a lower level in controls compared with cancer patients. In comparison between pre- and postoperative samples, the latter possessed less CD105/TGF*β*3 except for the two patients mentioned above whose CD105/TGF*β*3 levels were markedly elevated after operation (Table 3). No significant correlation was found between either TGF*β*3 or CD105/TGF*β*3 complexes and a Dukes' stage.

## DISCUSSION

In this study, we have evaluated the prognostic significance of intratumoral MVD identified using antibodies to CD105 or CD34. These data demonstrate that MVD quantified by only Mab to CD105 correlated with prognosis. Quantification of circulating CD105, its ligands TGF*β*1 and TGF*β*3 and the ligand–receptor complexes revealed elevated levels of CD105, TGF*β*3, CD105/TGF*β*3 and CD105/TGF*β*1 complexes but not TGF*β*1 in patients with colorectal cancer. Furthermore, the levels of CD105 were correlated with Dukes' stages. This study suggests that MVD examined using Mab to CD105 and the circulating levels of CD105 are of prognostic significance in patients with colorectal carcinoma.

That the MVD using CD34 was not correlated with prognosis raises important issues. It is not the first time that determination of MVD using a pan-endothelial marker has failed to be correlated with prognosis. For instance, in thyroid and laryngeal squamous cell carcinomas, MVD was not associated with disease-free or overall survival (Akslen and Livolsi, 2000; Pignataro *et al*, 2001). Furthermore, MVD was even positively correlated with better prognosis in renal cell carcinoma (Sabo *et al*, 2001). In both these studies, a Mab to CD34 was used to determine the MVD. There is an obvious difference between the reactivity of the two Mabs utilised in the present study. CD34 is an excellent marker for the normal vasculature, whereas CD105 antibody is more discriminatory in its staining of microvessels. By immunostaining of a variety of tissues, we and others have observed that whereas Mab to CD105 has a high affinity for angiogenic blood vessels (e.g. in tumours, stroke and psoriatic tissues), it often fails to react with the normal microvessels decorated by pan-endothelial markers, for example, CD31, vWF, CD34, PAL-E (Burrows *et al*, 1995; Kumar *et al*, 1996; Bodey *et al*, 1998; Miller *et al*, 1999; Brewer *et al*, 2000; Fonsatti *et al*, 2001 and our unpublished data). These observations demonstrate that CD105 and the pan-endothelial markers are differentially expressed in angiogenic and normal EC, and that the former is more suitable for identifying tumour angiogenesis. It is possible that colorectal cancers acquire their vasculature both by incorporation of normal vessels of the host (which are recognised by CD34 and other pan-endothelial markers) and induction of new blood vessels, that is, angiogenesis (recognised by CD105). The two sets of vasculature are thought to be functional in supporting tumour development, so why is the MVD recognised by Mab to CD105, but not by Mab to CD34, correlated with prognosis? It is possible that high microvessel counts identified by CD105 represent a high profile of angiogenesis in tumours that are more progressive and likely to metastasise. In contrast, MVD identified by CD34 (and other pan-endothelial markers) may not be able to accurately characterise angiogenesis of certain types of tumour, leading to a lack of association of MVD with tumour progression. This speculation is supported by a recent study by Akagi *et al* (2002), who have reported that a significant increase of MVD determined by Mab to CD105 but not Mab to CD34 was observed from low-grade to high-grade dysplasia of colorectal mucosa, and from high-grade dysplasia to colorectal carcinoma. In conclusion, the ability to quantitatively discriminate between angiogenesis and pre-existing vessels in tumours seems to be an important determinant in the assessment of tumour angiogenesis. Hence, MVD identified using Mab to CD105 proved superior over pan-endothelial markers such as CD34 in assessing the prognosis of patients with colorectal cancer.

With regard to the circulating levels of CD105, TGF*β*1, TGF*β*3 and the receptor–ligand complexes, the results indicate that except for TGF*β*1, the levels of the other molecules were markedly elevated in cancer patients compared with controls. Correlation analysis has revealed that CD105 is the only one that was positively correlated with a Dukes' stage. Furthermore, decreased levels of TGF*β*3 and CD105/TGF*β*3 complexes were noted in postoperative compared with preoperative samples. These observations strongly suggest that CD105, TGF*β*3 and CD105/TGF*β*3 complexes in the circulation may be of prognostic value in patients with colorectal cancer.

Raised levels of CD105 and TGF*β*3 were found in plasma samples from colorectal cancer patients, which are in agreement with previous observations (Li *et al*, 1998, Li *et al*, 2000b). Published data indicate that CD105 is strongly expressed in blood vessels in cancer tissues but weakly in normal tissues. Therefore, it is reasonable to assume that increased CD105 in the circulation of patients with cancer resulted from angiogenesis both within and in the immediate vicinity of the tumour mass. That CD105 levels were positively correlated with a Dukes' stage, was entirely consistent with the observation in patients with breast cancer, wherein significantly elevated CD105 levels were found in patients who subsequently relapsed or developed metastatic disease (Li *et al*, 2000b). A recent publication also supports the conclusion that patients with lymph node metastasis possess markedly elevated CD105 levels in the circulation (Takahashi *et al*, 2001). These data prompt us to propose that CD105 is an angiogenic marker that can be used for monitoring tumour metastasis and relapse.

Tumour cells produce TGF*β*1 and TGF*β*3 as shown in the current and previous studies (Ghellal *et al*, 2000). Their distinct tissue localisation in colorectal tumours implies that the two isoforms may orchestrate different biological functions in tumour development. In line with this speculation, TGF*β*3 but not TGF*β*1, has been found to be positively associated with lymph node metastasis in breast cancer patients and inversely correlated with the survival of patients with osteosarcoma (Kloen *et al*, 1997; Li *et al*, 1998), suggesting that modulating role of TGF*β*3 in tumour progression may differ in various tumour types. The underlying mechanism is not fully understood, but its proangiogenic action may contribute to the stimulating effects in tumour progression (Gold *et al*, 2000; Li *et al*, 2001). In this study TGF*β*3 levels were increased, although not significantly, as the disease progressed. The lack of a statistical significance is likely to be the result of having a relatively small number of patients in each group and a large variation in the TGF*β*3 levels. With respect to TGF*β*1, although published data show inconsistency, it is generally accepted that TGF*β*1 acts as a tumour suppressor, particularly at an early stage of the disease (Rich *et al*, 2001). A lower TGF*β*1 level was observed in patients with colorectal cancer compared with controls, which may have been resulted from the elevated CD105/TGF*β*1 complex levels in the same patients. The inverse correlation between TGF*β*1 levels and the number of positive lymph nodes indicates that TGF*β*1 may play a role in suppressing node metastasis, which is consistent with a previous observation (Gohongi *et al*, 1999).

To summarise, we have investigated the prognostic significance of the angiogenic marker CD105 and its ligands TGF*β*1 and TGF*β*3 in patients with colorectal cancer by immunohistochemistry and ELISAs. The conclusions are that a high MVD, identified using Mab to CD105, predicts a poor prognosis, and that circulating CD105, levels are positively correlated with Dukes' stage. Therefore, CD105, as a novel marker of tumour angiogenic activity, may prove to be valuable in assessing the prognosis of patients with colorectal cancer, especially in those patients who are receiving antiangiogenic therapies. Indeed our preliminary unpublished data show that the circulating levels of CD105 and CD105/TGF*β* complexes dramatically decreased in individuals who had been treated by a variety of antiangiogenic agents. These findings are part of an ongoing clinical trial and thus their precise clinical usefulness has not been evaluated.
